# The genome sequence of a robber fly, *Cyrtopogon pulchripes* Loew, 1871 (Diptera: Asilidae)

**DOI:** 10.12688/wellcomeopenres.28176.1

**Published:** 2026-10-23

**Authors:** Jaakko Pohjoismäki, Wellcome Sanger Institute Tree of Life Management, Samples and Laboratory team, Wellcome Sanger Institute Scientific Operations: Sequencing Operations, Wellcome Sanger Institute Tree of Life Core Informatics team, Tree of Life Core Informatics collective, Mark L. Blaxter

**Affiliations:** 1 University of Eastern Finland, Joensuu, North Karelia, Finland; 2 Tree of Life, Wellcome Sanger Institute, Hinxton, England, UK

**Keywords:** Cyrtopogon pulchripes, robber fly, genome sequence, chromosomal, Diptera

## Abstract

We present a genome assembly from an individual female *Cyrtopogon pulchripes* (robber fly; Arthropoda; Insecta; Diptera; Asilidae). The assembly contains two haplotypes with total lengths of 1,370.56 megabases and 1,360.19 megabases. Most of the haplotype 1 assembly (95.65%) was scaffolded into six chromosomal pseudomolecules. Haplotype 2 was assembled to scaffold level. The mitochondrial genome has also been assembled, with a length of 16.77 kilobases.

## Species taxonomy

Eukaryota; Opisthokonta; Metazoa; Eumetazoa; Bilateria; Protostomia; Ecdysozoa; Panarthropoda; Arthropoda; Mandibulata; Pancrustacea; Altocrustacea; Allotriocarida; Hexapoda; Insecta; Dicondylia; Pterygota; Neoptera; Eumetabola; Endopterygota; Aparaglossata; Panorpida; Diptera; Brachycera; Muscomorpha; Asiloidea; Asilidae; Brachyrhopalinae; *Cyrtopogon*; *Cyrtopogon pulchripes* Loew, 1871 (NCBI:txid2881815)

## Background

*Cyrtopogon pulchripes* Loew, 1871 is a member of the family Asilidae, commonly known as robber flies or assassin flies. The family Asilidae is one of the largest groups of true flies, with more than 7,000 described species globally, and constitutes a diverse and widespread lineage within the asiloid flies ( Dikow, 2009). *Cyrtopogon* is a Holarctic genus of robber flies whose species frequently exhibit pronounced sexual dimorphism, with males often possessing striking ornamental colouration and leg modifications that are likely associated with sexual signalling and mate recognition ( Wolff *et al.*, 2018). *Cyrtopogon pulchripes* is an exceptionally rare eastern Palaearctic species that is known in Europe only from Finland ( Engel, 1930; Pohjoismäki *et al.*, 2023; Richter, 1988; Tagawa, 2001).

Adults of *Cyrtopogon pulchripes* are medium-sized robber flies measuring 10–15 mm in body length, with a predominantly black and shiny appearance combined with contrasting yellowish leg markings (Figure 1) ( Pohjoismäki *et al.*, 2023). Males are readily recognised by the large triangular yellow-dusted patch on abdominal tergite 2, whereas females possess narrow grey transverse bands on tergites 2 to 5. Legs are characteristically bicoloured, with black femora and yellowish tibiae that darken apically, giving rise to the species name *pulchripes* (“beautiful-legged”). Adults have been recorded in Finland from June to August, but the species is probably most detectable around midsummer.

*Cyrtopogon pulchripes* is a very local and rarely encountered species in eastern Finland ( Pohjoismäki *et al.*, 2023). The species has an eastern Palaearctic distribution, with European records restricted to Finland, where it occurs sporadically in inland eastern regions. Remarkably, the species was thought possibly extinct in Finland after the last confirmed observation in Kuopio in 1916. It was rediscovered in 2012 at Korkeakoski, Maaninka, a locality from which the species had previously been recorded in 1865. Additional records from Liperi subsequently confirmed the persistence of at least one extant population, including the first observations of females since 1916.

The species inhabits warm and sheltered conifer-dominated mixed forest edges with exposed mineral soil ( Pohjoismäki *et al.*, 2023). Habitat observations suggest an association with ecotones between mixed forests and open habitats such as railway margins or agricultural clearings. Although previously regarded as an old-growth forest species, recent observations indicate that it may persist in relatively ordinary but structurally diverse managed forests when suitable sandy microhabitats are present. Males have been observed resting in sunlit patches within forests and have also been collected using coloured pan traps. Females have been seen sitting on bare sandy ground and ovipositing at the base of heather growing in sandy substrates. Known prey items include flies, mosquitoes, microlepidoptera, and small hymenopterans.

Due to its rarity, patchy occurrence, and elusive behaviour, the biology of *Cyrtopogon pulchripes* remains poorly understood ( Pohjoismäki *et al.*, 2023). Despite intensive searches, some historical populations have not been relocated, suggesting that the species may occur at low population densities or have highly specific habitat requirements. In the Finnish Red List assessment of 2019, the species was classified as Near Threatened (NT) ( Haarto *et al.*, 2019).

The reference genome assembly of *C. pulchripes* provides an important genomic resource for Asilidae and contributes to the expanding genomic representation of robber flies. It offers opportunities to investigate their population genetics, evolution and physiological adaptations.

**Figure 1.**
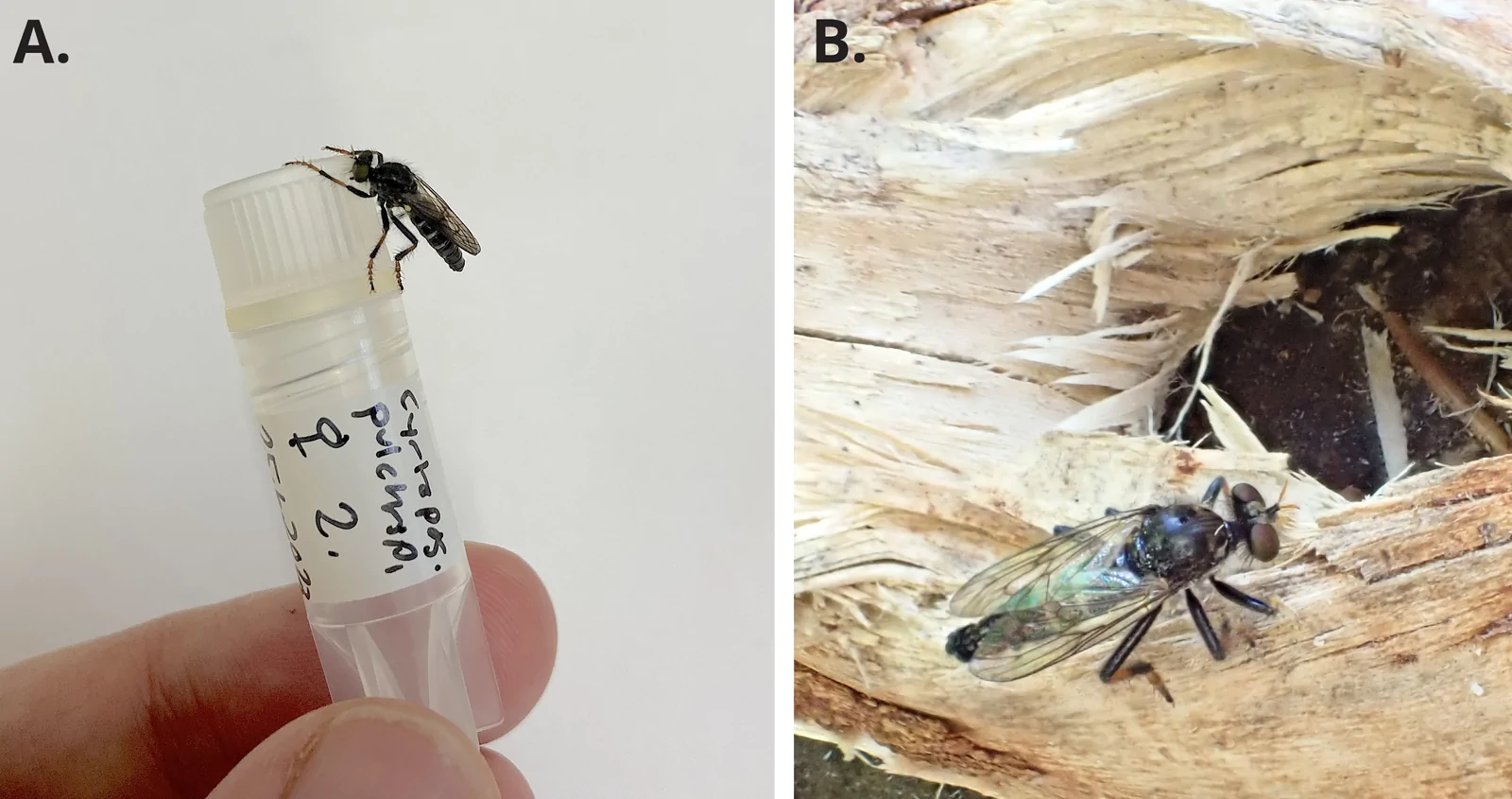
*Cyrtopogon pulchripes*. A. A female specimen (idCyrPulc1) collected for genome sequencing. B. A male specimen from the same locality, not collected.

## Methods

### Sample acquisition

The specimen used for genome sequencing was an adult female *Cyrtopogon pulchripes* (specimen ID SAN00003431, ToLID idCyrPulc1; Figure 1, panel A). The same specimen was also used for Hi-C sequencing. It was collected from Hautalahti, Liperi, North Karelia, Finland (latitude 62.5574, longitude 29.1049) on 2023-06-25 by Jaakko Pohjoismaki (University of Eastern Finland), who also identified it.

### Nucleic acid extraction

Detailed protocols for nucleic acid extraction developed at the Wellcome Sanger Institute (WSI) Tree of Life Core Laboratory are available on protocols.io ( Howard *et al.*, 2025). The idCyrPulc1 specimen was weighed and triaged to determine the appropriate extraction protocol.

For high molecular weight (HMW) DNA extraction, 24.0 mg of whole organism tissue was homogenised by PowerMash using a PowerMasher II tissue disruptor. HMW DNA was extracted using the Automated MagAttract v2 protocol. The DNA was fragmented following Sanger Tree of Life HMW DNA Fragmentation: Diagenode Megaruptor3 for LI PacBio. Sheared DNA was purified by automated SPRI (solid-phase reversible immobilisation).

The concentration of the sheared and purified DNA was assessed with a NanoDrop spectrophotometer and a Qubit Fluorometer using the Qubit dsDNA High Sensitivity Assay kit. Fragment size distribution was evaluated by running the sample on the Femto Pulse system. For this sample, final post-shearing DNA measurements included a Qubit concentration of 37.93 ng/μL, a yield of 1,782.71 ng, and an average fragment size of 16.8 kb.

### PacBio HiFi library preparation and sequencing

Library DTOL14901120 was prepared with the SMRTbell Prep Kit 3.0 (Pacific Biosciences) according to the manufacturer’s instructions. The kit includes reagents for end repair/A-tailing, adapter ligation, post-ligation SMRTbell bead clean-up, and nuclease treatment. Size selection and clean-up were performed using diluted AMPure PB beads (Pacific Biosciences). DNA concentration was quantified with a Qubit Fluorometer v4.0 (Thermo Fisher Scientific) and the Qubit 1X dsDNA HS assay kit. Final library fragment size was assessed with the Agilent Femto Pulse Automated Pulsed Field CE Instrument (Agilent Technologies) using the gDNA 55 kb BAC analysis kit.

The library was sequenced on a Revio instrument (Pacific Biosciences, California, USA). Prepared libraries with a molarity greater than 2 nM were normalised to 2 nM, and 15 μL was used to make complexes. For libraries with a molarity below 2 nM, the available 10 μL library volume was used without normalisation. Primers were annealed and polymerases were bound to generate circularised complexes following the manufacturer’s instructions. Complexes were purified using 1.2× SMRTbell beads, diluted to a loading concentration of 200–300 pM, and spiked with a Revio sequencing internal control. Sequencing used Revio 25M SMRT cells. SMRT Link software was used to configure and monitor the run and to perform primary and secondary data analysis.

### Hi-C

***Sample preparation and crosslinking***

Hi-C data were generated from the idCyrPulc1 whole organism sample using the Arima-HiC v2 kit (Arima Genomics). Following the manufacturer’s instructions, tissue was fixed and DNA crosslinked using TC buffer to a final formaldehyde concentration of 2%. The tissue was homogenised using the Diagnocine Power Masher-II. Crosslinked DNA was digested with a restriction enzyme master mix, biotinylated, and ligated. Clean-up was performed with SPRISelect beads before library preparation. DNA concentration was measured with the Qubit Fluorometer (Thermo Fisher Scientific) and Qubit HS Assay Kit. The biotinylation percentage was estimated using the Arima-HiC v2 QC beads.

***Hi-C library preparation and sequencing***

Biotinylated DNA constructs were fragmented using a Covaris E220 sonicator and size-selected to 400–600 bp using SPRISelect beads. DNA was enriched with Arima-HiC v2 kit Enrichment beads. End repair, A-tailing, and adapter ligation were carried out with the NEBNext Ultra II DNA Library Prep Kit (New England Biolabs), following a modified protocol where library preparation occurs while DNA remains bound to the Enrichment beads. Libraries were amplified with KAPA HiFi HotStart mix and a custom Unique Dual Index (UDI) barcode set (Integrated DNA Technologies). Depending on sample concentration and biotinylation percentage determined at the crosslinking stage, libraries were amplified with 10–16 PCR cycles. Post-PCR clean-up was performed with SPRISelect beads. Libraries were quantified using the AccuClear Ultra High Sensitivity dsDNA Standards Assay Kit (Biotium) and a FLUOstar Omega plate reader (BMG Labtech).

Prior to sequencing, libraries were normalised to 10 ng/μL. Normalised libraries were quantified again to create equimolar and/or weighted 2.8 nM pools. Pool concentrations were checked using the Agilent 4200 TapeStation (Agilent) with High Sensitivity D500 reagents before sequencing. Libraries were sequenced on the Illumina NovaSeq X, generating paired-end 150-bp reads.

### Genome assembly

Prior to assembly of the PacBio HiFi reads, a database of *k*-mer counts ( *k* = 31) was generated from the filtered reads using FastK. The *k*-mer frequency distribution was analysed with GenomeScope (version 2.1.0) ( Ranallo-Benavidez *et al.*, 2020), providing fitted point estimates of haploid genome size, heterozygosity and repeat content.

The HiFi reads were assembled using Hifiasm in Hi-C phasing mode ( Cheng *et al.*, 2021, 2022), producing two haplotypes. Hi-C reads ( Rao *et al.* , 2014) were mapped to contigs using bwa-mem2 ( Vasimuddin *et al.*, 2019). Contigs were further scaffolded with Hi-C data in YaHS ( Zhou *et al.*, 2023), using the --break option for handling potential misassemblies. The scaffolded assemblies were evaluated using Gfastats ( Formenti *et al.* , 2022), BUSCO ( Tegenfeldt *et al.*, 2025) and MerquryFK ( Rhie *et al.*, 2020).

The mitochondrial genome was assembled using MitoHiFi ( Uliano-Silva *et al.* , 2023). The MitoHiFi reference was the mitochondrial genome of *Dasypogon diadema* (NC_045239.1).

### Assembly curation

The assembly was decontaminated using the Assembly Screen for Cobionts and Contaminants ( ASCC) pipeline. The flat files and maps for curation were generated with TreeVal. Manual curation was conducted primarily in PretextView and HiGlass ( Kerpedjiev *et al.* , 2018). Scaffolds were visually inspected and corrected as described by Howe *et al*. (2021). The two haplotype assemblies were combined for curation. Manual corrections included 39 breaks and 67 joins. This reduced the scaffold count by 4.0% and reduced the scaffold N50 by 36.4%. The curation process is described at sanger-tol/curation-resources. A Hi-C contact map of the haplotype 1 assembly was generated with PretextSnapshot.

### Assembly quality assessment

The MerquryFK tool ( Rhie *et al.*, 2020) was run in a Singularity container ( Kurtzer *et al.*, 2017) to evaluate *k*-mer completeness and assembly quality for both haplotypes using the *k*-mer database ( *k* = 31) computed prior to genome assembly. The analysis outputs included assembly QV scores and completeness statistics.

The haplotype 1 assembly was assessed using BlobToolKit ( Challis *et al.*, 2020). PacBio reads were mapped to the assembly to derive sequence coverage, and BUSCO ( Tegenfeldt *et al.*, 2025) was used to assess gene-set completeness. DIAMOND BLASTp searches of extracted BUSCO proteins and DIAMOND BLASTx searches of assembly sequences against UniProt Reference Proteomes ( Buchfink *et al.*, 2021), followed by BLASTn searches of sequences without DIAMOND BLASTx hits against the NCBI nt database ( Altschul *et al.*, 1990), supported taxonomic assignment. Coverage, sequence composition, taxonomic assignment and BUSCO results were integrated into a BlobDir for interactive inspection and visualisation. The snail and blob plots were rendered locally from this hosted BlobDir using BlobTk v0.8.3. The scaffold-length radial scale function for the snail plot was set to linear ( Challis & Blaxter, 2026). The blob plot used circular markers with area proportional to scaffold length ( Challis *et al.*, 2020).

Ancestral linkage groups were painted along the chromosome-level scaffolds of the haplotype 1 assembly. Complete BUSCO genes (single-copy and duplicated) from the diptera_odb12 lineage dataset were assigned to the 10 Brachycera ancestral linkage groups (db1a–db4b, db5 and db6) described by Gries *et al.*. (2026) and plotted along chromosomes drawn to scale.

## Genome sequence report

### Sequence data

PacBio sequencing of the *C. pulchripes* specimen generated 35.53 Gb (gigabases) from 3.41 million reads. These reads were used to assemble the genome. GenomeScope analysis using a *p* = 2 model gave a *k*-mer-based haploid genome size estimate of 1,315.72 Mb, with heterozygosity of 1.02% and repeat content of 44.94%; the full-model fit was 98.64% (Figure 2). The GenomeScope estimates guided expectations for the assembly. The NCBI assembly record reports 26× genome coverage. Hi-C sequencing produced 119.25 Gb from 394.87 million read pairs, which were used to scaffold the haplotype 1 assembly. Table 1 summarises the specimen and sequencing details.

**Figure 2.**
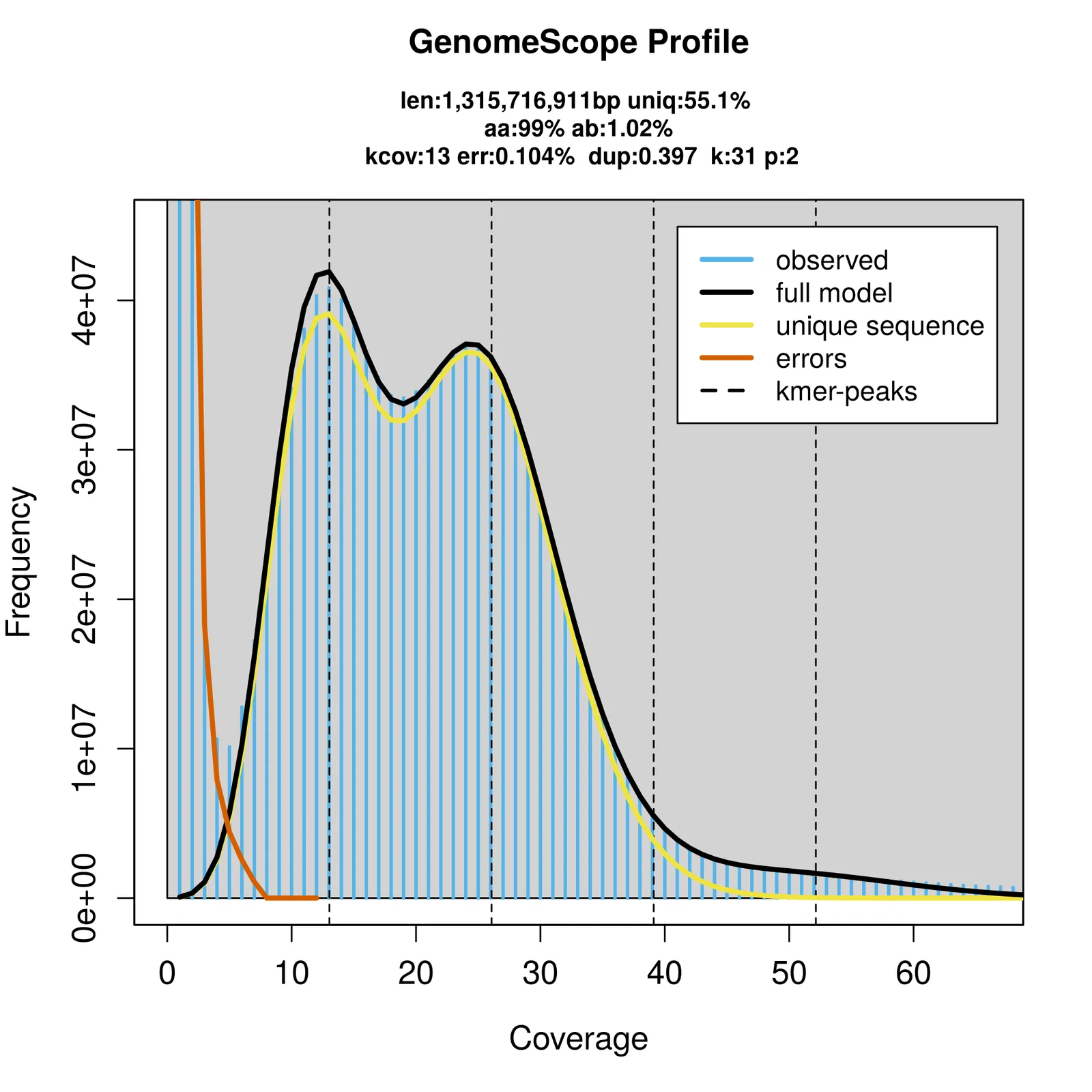
GenomeScope model fitted to the *k*-mer frequency distribution. The plot shows the observed and modelled *k*-mer spectra used to estimate haploid genome size, heterozygosity and repeat content from unassembled sequencing reads.

**Table 1.** Specimen and sequencing data for *C. pulchripes* (BioProject PRJEB80967)

**Platform**	**PacBio HiFi**	**Hi-C**
**ToLID**	idCyrPulc1	idCyrPulc1
**Specimen ID**	SAN00003431	SAN00003431
**BioSample (source individual)**	SAMEA114663811	SAMEA114663811
**BioSample (tissue)**	SAMEA114663821	SAMEA114663821
**Tissue**	whole organism	whole organism
**Instrument**	Revio	Illumina NovaSeq X
**Library ID & run accession(s)**	DTOL14901120: ERR13800504	ERX13203398: ERR13802646
**Read count total**	3.41 million reads	394.87 million read pairs
**Base count total**	35.53 Gb	119.25 Gb

### Assembly statistics

The genome was assembled into two haplotypes using Hi-C phasing. The haplotype 1 assembly was curated to chromosome level, while haplotype 2 was assembled to scaffold level. The haplotype 1 assembly, which is proposed as the reference assembly for this species, has a total length of 1,370.56 Mb in 757 scaffolds (excluding organelle sequences), with 1,089 gaps, and its scaffold N50 is 237.73 Mb (Table 2). The scaffold auN for the haplotype 1 assembly is 265 Mb.

**Table 2.** Genome assembly statistics for *C. pulchripes*

**Genome assembly**	**Haplotype 1**	**Haplotype 2**
**Assembly name**	idCyrPulc1.hap1.1	idCyrPulc1.hap2.1
**Assembly accession**	GCA_971800845.1	GCA_971793335.1
**Assembly level**	chromosome	scaffold
**Span (Mb)**	1,370.56	1,360.19
**Number of chromosomes**	6	-
**Number of contigs**	1,846	1,511
**Contig N50 (Mb)**	2.18	2.13
**Number of scaffolds (excluding organelle sequences)**	757	454
**Scaffold N50 (Mb)**	237.73	238.11
**Scaffold auN (Mb)**	265	264
**Longest scaffold length (Mb)**	374.32	375.8
**Organelles**	Mitochondrial genome: 16.77 kb	-

Most of the haplotype 1 assembly sequence (95.65%) was assigned to 6 chromosome-level scaffolds. These chromosome-level scaffolds, confirmed by Hi-C data, are named according to size (Figure 3; Table 3). We did not identify the sex chromosomes because sequence data from the heterogametic sex were unavailable, and frequent sex chromosome turnover in Diptera makes identification by homology unreliable ( Vicoso & Bachtrog, 2015). During curation, we noted that scaffolds in the following regions have uncertain order and orientation: Chromosome 3 ~86.45–88.62 Mb, Chromosome 4 ~57.55–58.18 Mb and Chromosome 6 ~7.11–7.90 Mb. A large number of centromeric repeats remain in the unlocalised sequences.

The mitochondrial genome was also assembled (length 16.77 kb, OZ310517.1). This sequence is included as a contig in the multifasta file of the haplotype 1 genome submission and as a standalone record.

**Figure 3.**
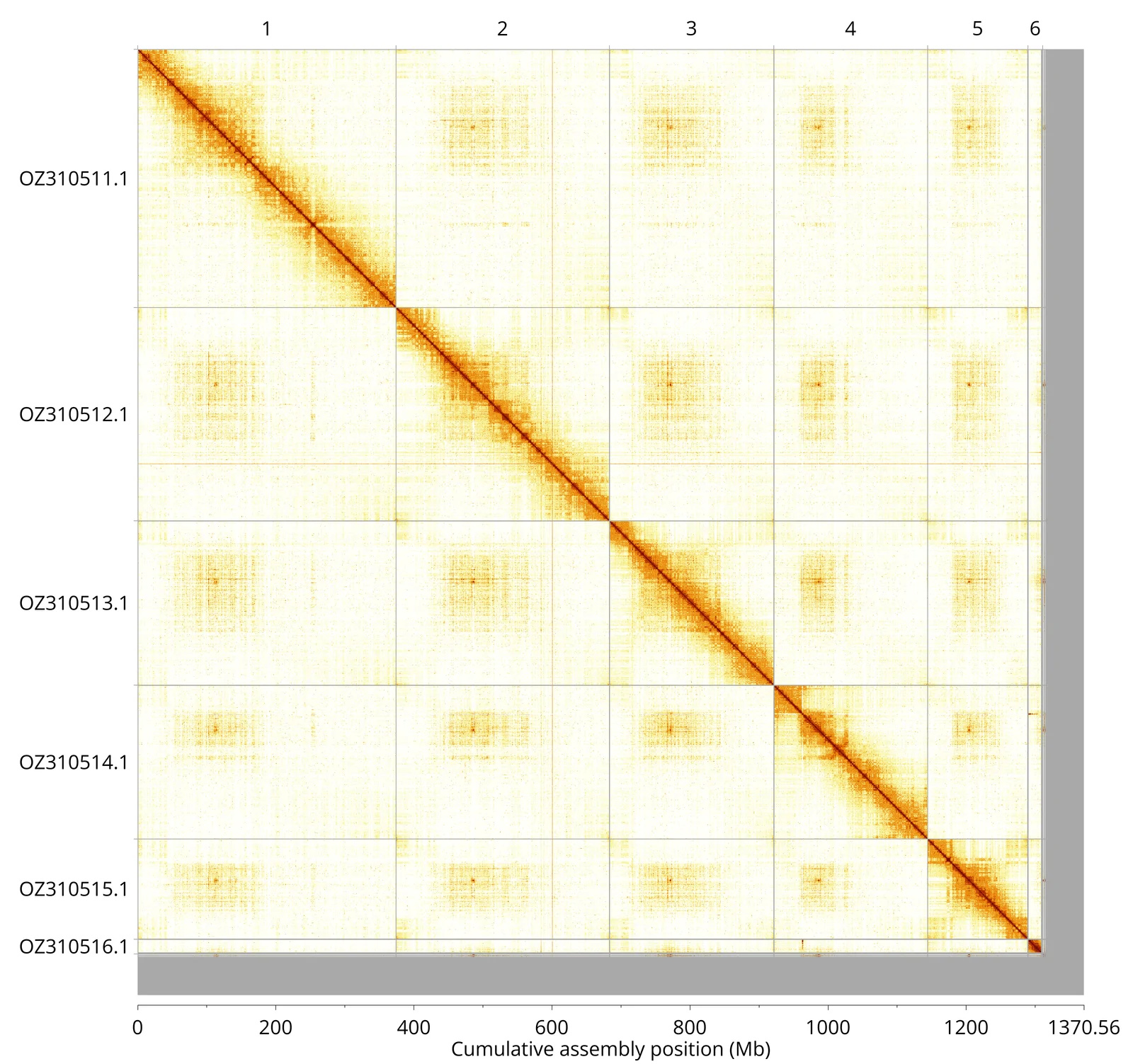
Hi-C contact map of the *C. pulchripes* haplotype 1 assembly. Assembled chromosomes are shown in order of size and labelled along the axes, with a megabase scale shown below. The plot was generated using PretextSnapshot.

**Table 3.** Chromosomal pseudomolecules in the haplotype 1 genome assembly of *C. pulchripes* idCyrPulc1

**INSDC accession**	**Molecule**	**Length (Mb)**	**GC%**
OZ310511.1	1	374.32	37.50
OZ310512.1	2	309.38	37.50
OZ310513.1	3	237.98	38
OZ310514.1	4	222.69	37.50
OZ310515.1	5	145.38	37.50
OZ310516.1	6	21.20	37.50

Chromosome painting shows the distribution of BUSCO genes assigned to Brachycera ancestral linkage groups across the chromosome-level scaffolds of the haplotype 1 assembly (Figure 4).

**Figure 4.**
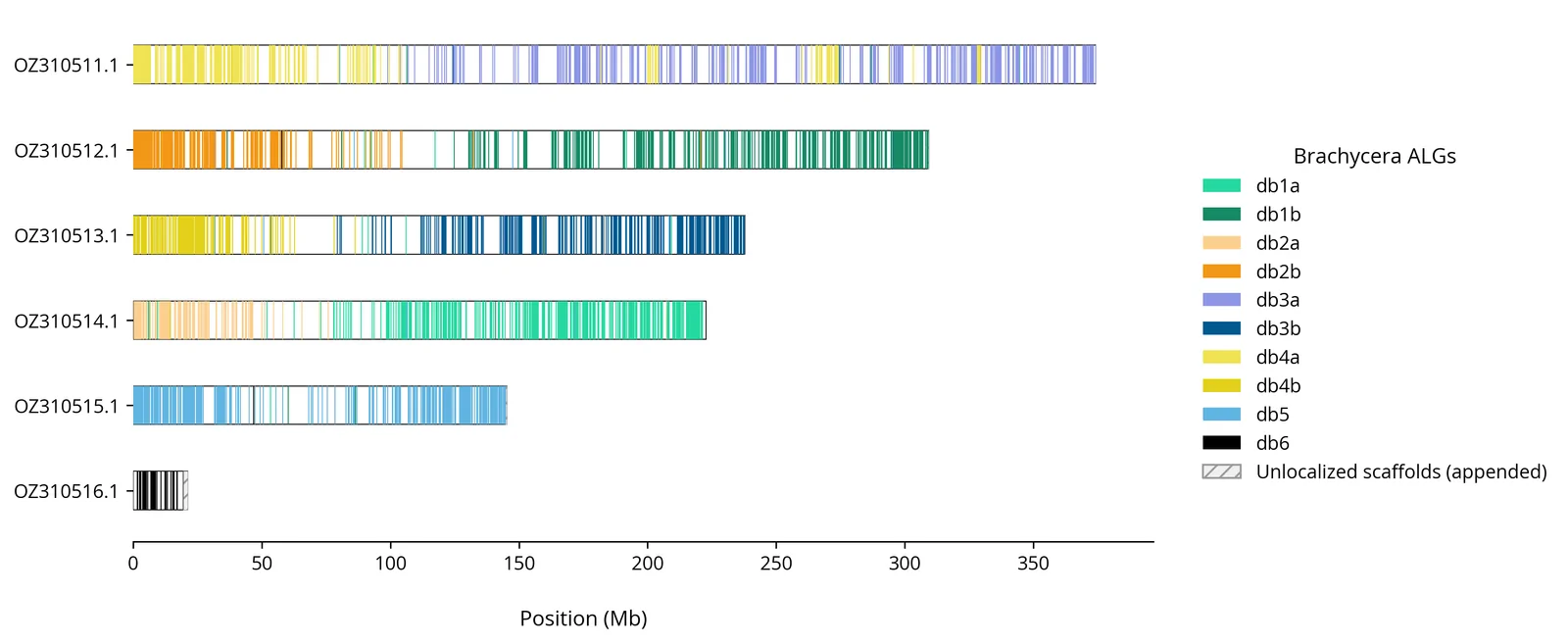
Brachycera ancestral linkage groups painting of the haplotype 1 assembly of *C. pulchripes*. Chromosomes are drawn to scale, with coloured bars marking the positions of complete diptera_odb12 BUSCO genes. Each BUSCO gene is coloured by its assignment to one of the 10 Brachycera ALGs (db1a–db4b, db5 and db6).

### Assembly quality metrics

For the haplotype 1 assembly, the estimated QV is 59.1, and for the haplotype 2 assembly, 59.2. When the two haplotypes are combined, the assembly achieves an estimated QV of 59.1. The *k*-mer completeness is 81.14% for the haplotype 1 assembly, 81.07% for the haplotype 2 assembly, and 99.09% for the combined haplotypes (Figure 5).

**Figure 5.**
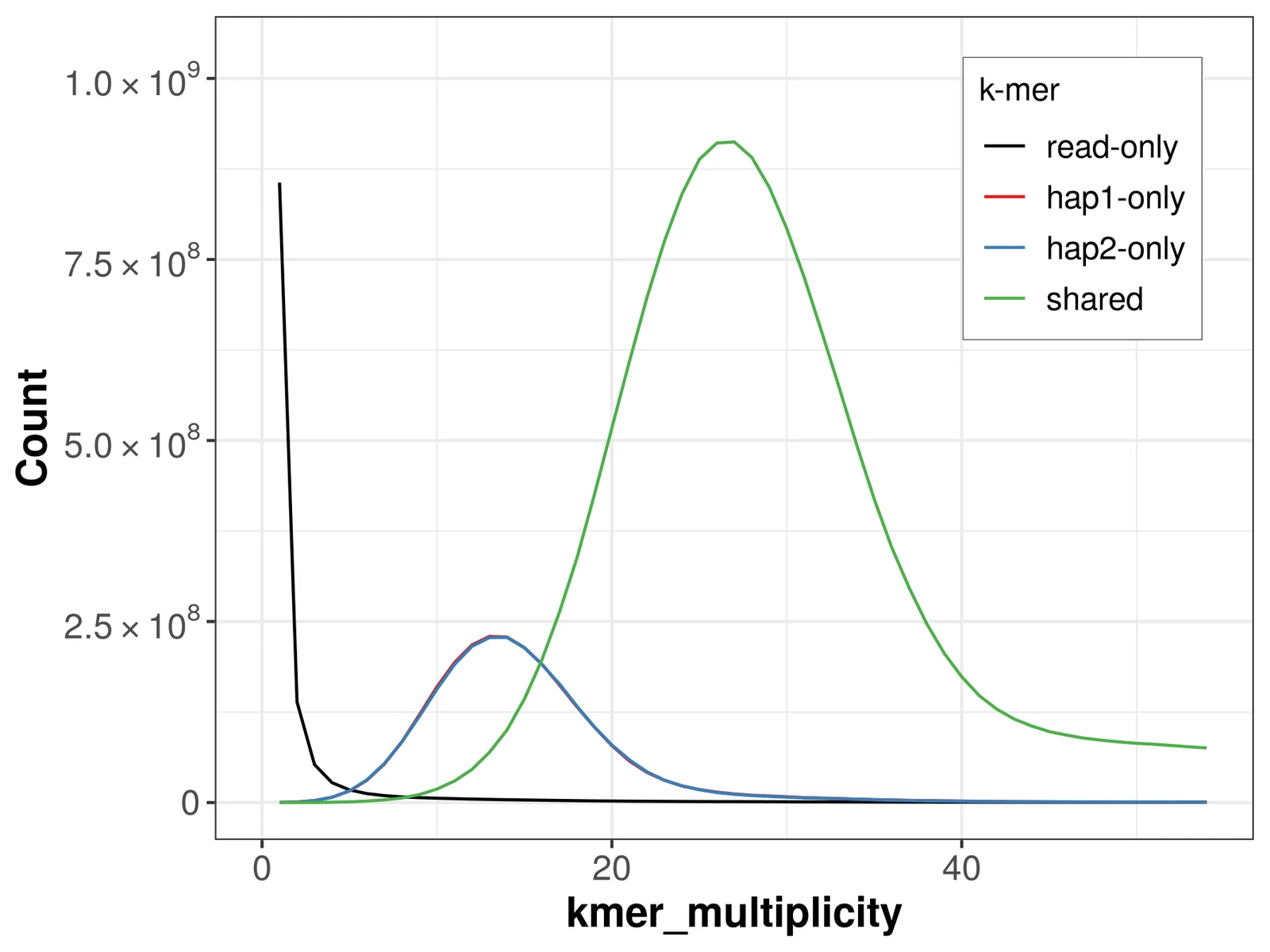
Evaluation of *k*-mer completeness using MerquryFK. This plot illustrates the recovery of *k*‐mers from the original read data in the haplotype 1 assembly, the haplotype 2 assembly and the combined haplotypes. The horizontal axis represents *k*‐mer multiplicity, and the vertical axis shows the number of *k*‐mers. The black curve represents *k*‐mers that appear in the reads but are not assembled. The green curve corresponds to *k*‐mers shared by both haplotypes, and the red and blue curves show *k*‐mers found only in one of the haplotypes.

BUSCO analysis using the diptera_odb10 reference set ( *n* = 3,285) identified 97.7% of the expected gene set (single = 95.8%, duplicated = 1.9%) in the haplotype 1 assembly. For the haplotype 2 assembly, BUSCO v.6.0.0 analysis identified 97.6% of the expected gene set (single = 95.8%, duplicated = 1.8%).

The snail plot in Figure 6 provides a summary of assembly contiguity, cumulative scaffold count, sequence composition and BUSCO completeness for the haplotype 1 assembly ( Challis & Blaxter, 2026). The relative sizes of the longest scaffold, scaffold N50 and N90, together with the dark-grey scaffold-length distribution, show how much of the assembly is contained in long scaffolds. The outer composition track shows variation in GC, AT and N content, while the central track shows the cumulative scaffold count and the BUSCO inset summarises gene-set completeness.

The blob plot in Figure 7 displays scaffolds from the haplotype 1 assembly by GC proportion and base coverage. Marker area is proportional to scaffold length, and colour indicates taxonomic assignment ( Challis *et al.*, 2020).

**Figure 6.**
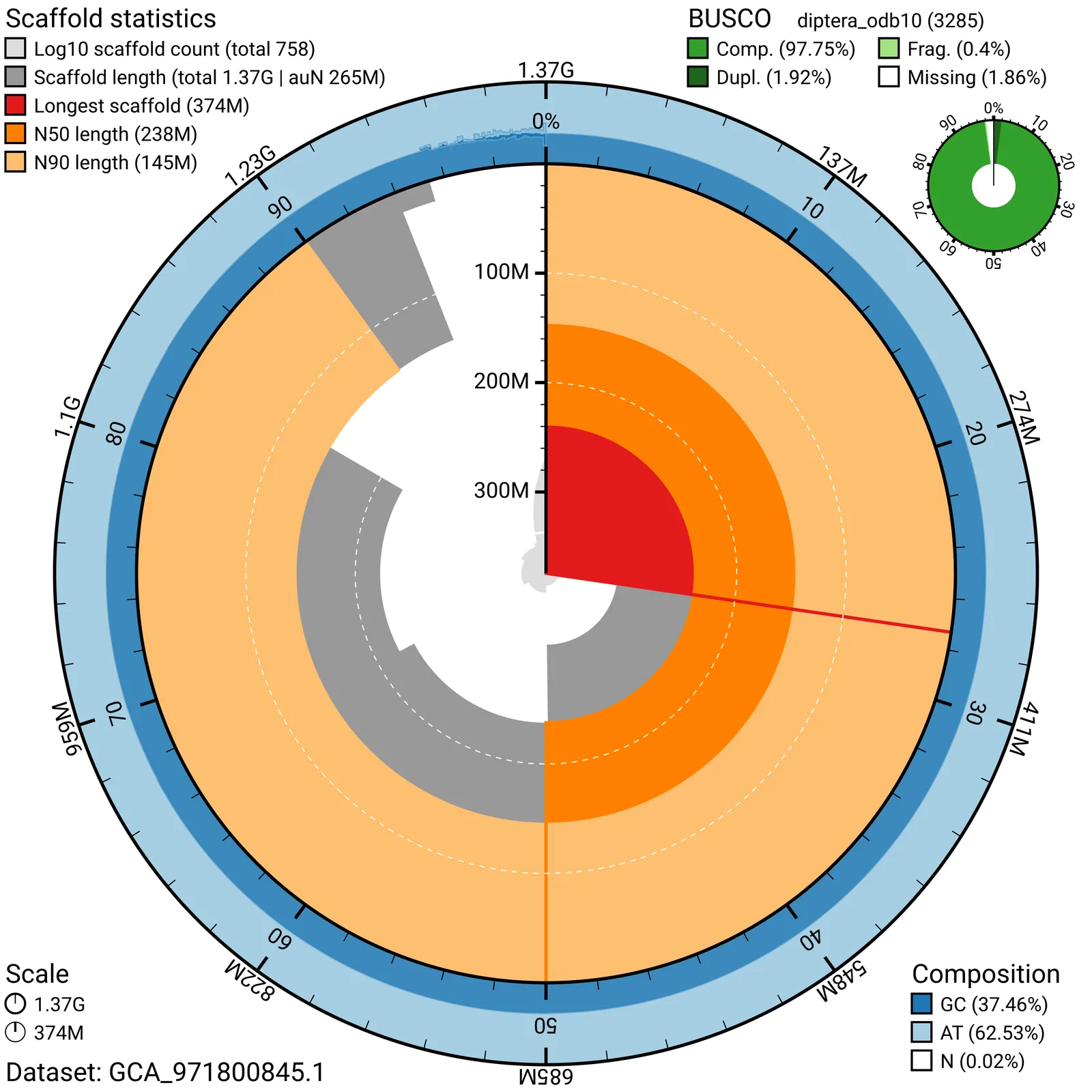
Assembly metrics for idCyrPulc1.hap1.1, the haplotype 1 assembly. The BlobToolKit snail plot provides an overview of assembly metrics and BUSCO gene completeness. The circumference represents the length of the haplotype 1 assembly, and the main plot is divided into 1,000 bins around the circumference. The outermost blue tracks display the distribution of GC, AT, and N percentages across the bins. Scaffolds are arranged clockwise from longest to shortest and are depicted in dark grey. Scaffold length is shown on a linear radial scale, scaled to the longest scaffold. Red shading indicates the longest scaffold, and the deeper orange and pale orange shading represent the N50 and N90 lengths. The summary statistics include scaffold auN, the area under the scaffold Nx curve. The light grey spiral at the centre shows the cumulative scaffold count on a logarithmic scale. A summary of complete, fragmented, duplicated, and missing BUSCO genes in the diptera_odb10 set is presented at the top right. The assembly can be explored interactively in the BlobToolKit viewer.

**Figure 7.**
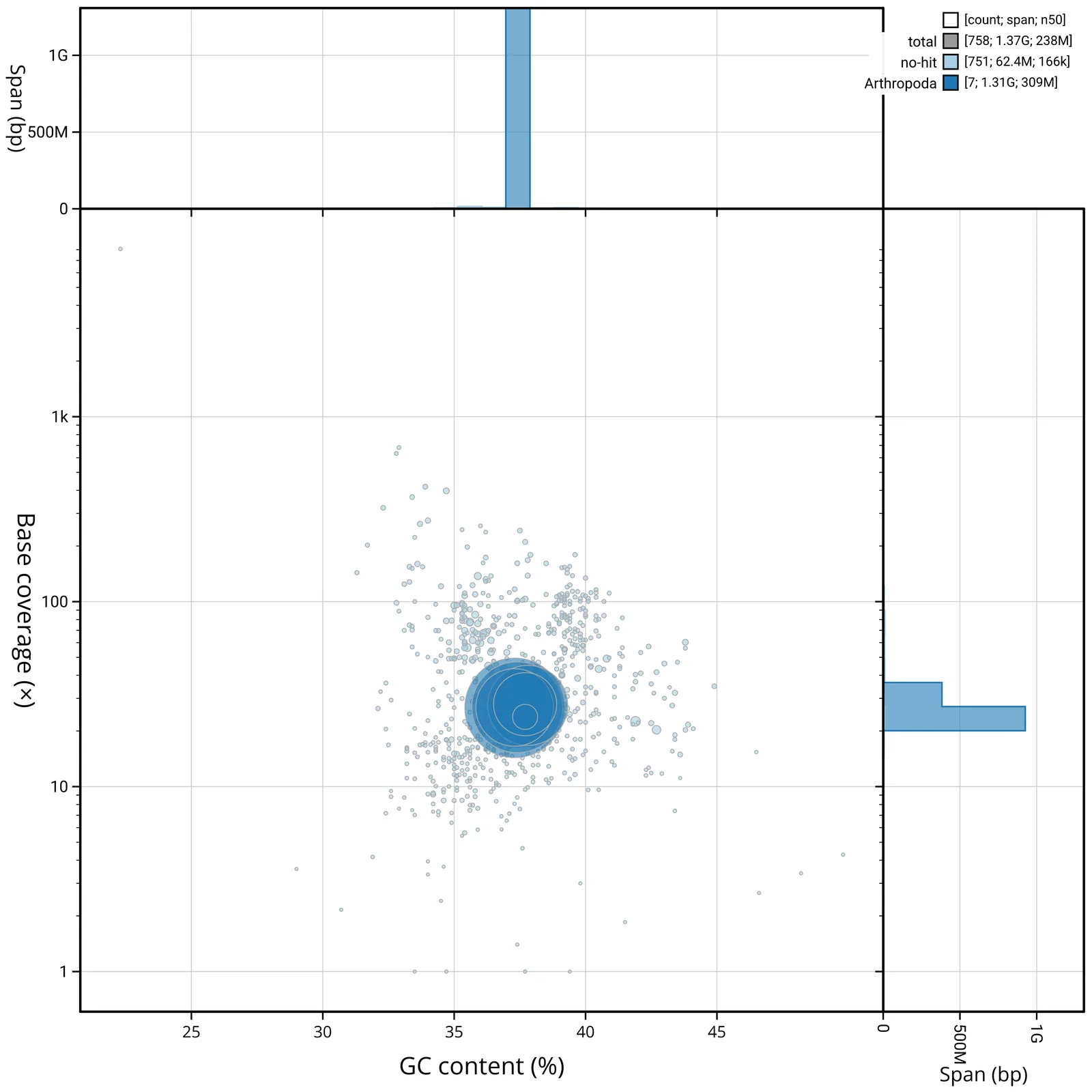
BlobToolKit blob plot for idCyrPulc1.hap1.1, the haplotype 1 assembly. The plot shows base coverage (vertical axis) and GC content (horizontal axis). Circles represent scaffolds; marker area is proportional to scaffold length, and colour indicates taxonomic assignment. The histograms along the axes display the total length of sequences distributed across different levels of coverage and GC content. The assembly can be explored interactively in the BlobToolKit viewer.

Table 4 presents assembly quality metrics alongside the Earth BioGenome Project (EBP) benchmarks described by Howe *et al.*. (2026). The three-part EBP metric, calculated for the haplotype 1 assembly, is **6.C.Q59**.

**Table 4.** Earth BioGenome Project summary metrics for the *C. pulchripes* assembly

**Measure**	**Value**	***Benchmark***
EBP summary (haplotype 1)	6.C.Q59	*6.C.Q40*
Contig N50 length	2.18 Mb	*> 1 Mb*
Scaffold N50 length	237.73 Mb	*= chromosome N50*
Consensus quality (QV)	Haplotype 1: 59.1; haplotype 2: 59.2; combined: 59.1	*≥ 40*
*k*-mer completeness	Haplotype 1: 81.14%; Haplotype 2: 81.07%; combined: 99.09%	*> 90% (combined assembly)*
BUSCO	Haplotype 1: C:97.7% [S:95.8%, D:1.9%], F:0.4%, M:1.9%, n:3,285; Haplotype 2: C:97.6% [S:95.8%, D:1.8%], F:0.6%, M:1.8%, n:3,285	*S > 90%*
Percentage of assembly assigned to chromosomes	95.65%	*≥ 90%*

**Notes:** The EBP summary uses log10(Contig N50); chromosome-level (C) or log10(Scaffold N50); Q (Merqury QV). For *k*-mer completeness, the benchmark is assessed using the combined assembly value. BUSCO v6.0.0 using the diptera_odb10 lineage dataset: C=complete; S=single-copy; D=duplicated; F=fragmented; M=missing; n=orthologues.

### Data availability

European Nucleotide Archive: Cyrtopogon pulchripes. Accession number PRJEB80967; https://identifiers.org/ena.embl/PRJEB80967. The genome sequence is released openly for reuse. The *C. pulchripes* genome sequencing initiative is part of the Sanger Institute Tree of Life Programme ( PRJEB43745). All raw sequence data and the haplotype assemblies have been deposited in INSDC databases. The genome will be annotated using available RNA-Seq data and presented through the Ensembl pipeline at the European Bioinformatics Institute. Raw data and assembly accession identifiers are reported in Table 1 and Table 2.

Production code used in genome assembly at the WSI Tree of Life is available at https://github.com/sanger-tol. Table 5 lists software versions used in this study.

**Table 5.** Software versions and sources

**Software**	**Version**	**Source**
BLAST	2.14.0	https://ftp.ncbi.nlm.nih.gov/blast/executables/blast+/
BlobToolKit	4.4.6	https://github.com/genomehubs/blobtoolkit
BlobTk (hosted BlobDir)	0.5.1	https://github.com/genomehubs/blobtk
BlobTk (snail and blob plot renderer)	0.8.3	https://github.com/genomehubs/blobtk
BUSCO	6.0.0	https://gitlab.com/ezlab/busco
busco-alg-painter	0.1.0	https://github.com/Karenvn/busco-alg-painter
bwa-mem2	2.2.1	https://github.com/bwa-mem2/bwa-mem2
DIAMOND	2.1.8	https://github.com/bbuchfink/diamond
fasta_windows	0.2.4	https://github.com/tolkit/fasta_windows
FastK	1.2	https://github.com/thegenemyers/FASTK
GenomeScope	2.1.0	https://github.com/tbenavi1/genomescope2.0
Gfastats	1.3.6	https://github.com/vgl-hub/gfastats
Hifiasm	0.19.8-r603	https://github.com/chhylp123/hifiasm
HiGlass	1.13.4	https://github.com/higlass/higlass
MerquryFK	1.1.0-c1	https://github.com/thegenemyers/MERQURY.FK
Minimap2	2.28-r1209	https://github.com/lh3/minimap2
MitoHiFi	3.2.2	https://github.com/marcelauliano/MitoHiFi
MultiQC	1.14; 1.17 and 1.18	https://github.com/MultiQC/MultiQC
Nextflow	24.10.4	https://github.com/nextflow-io/nextflow
PretextSnapshot	0.0.6	https://github.com/sanger-tol/PretextSnapshot
PretextView	1.0.3	https://github.com/sanger-tol/PretextView
samtools	1.21	https://github.com/samtools/samtools
sanger-tol/ascc	0.1.0	https://github.com/sanger-tol/ascc
sanger-tol/blobtoolkit	v0.9.0	https://github.com/sanger-tol/blobtoolkit
sanger-tol/curationpretext	1.4.2	https://github.com/sanger-tol/curationpretext
Seqtk	1.4-r122	https://github.com/lh3/seqtk
Singularity	3.9.0	https://github.com/sylabs/singularity
TreeVal	1.1.1	https://github.com/sanger-tol/treeval
YaHS	1.2a.2	https://github.com/c-zhou/yahs

### Author information

Contributors are listed at the following links:

- Members of the Wellcome Sanger Institute Tree of Life Management, Samples and Laboratory team
- Members of Wellcome Sanger Institute Scientific Operations – Sequencing Operations
- Members of the Wellcome Sanger Institute Tree of Life Core Informatics team
- Members of the Tree of Life Core Informatics collective

### Wellcome Sanger Institute – Legal and Governance

The materials that have contributed to this genome note have been supplied by a Tree of Life collaborator. The Wellcome Sanger Institute employs a process whereby due diligence is carried out proportionate to the nature of the materials themselves, and the circumstances under which they have been/are to be collected and provided for use. The purpose of this is to address and mitigate any potential legal and/or ethical implications of receipt and use of the materials as part of the research project, and to ensure that in doing so we align with best practice wherever possible.

The overarching areas of consideration are:

- Ethical review of provenance and sourcing of the material
- Legality of collection, transfer and use (national and international)

Each transfer of samples is undertaken according to a Research Collaboration Agreement or Material Transfer Agreement entered into by the Tree of Life collaborator, Genome Research Limited (operating as the Wellcome Sanger Institute) and in some circumstances other Tree of Life collaborators.
