# Supplementary material for: The genome sequence of a robber fly, Cyrtopogon pulchripes Loew, 1871 (Diptera: Asilidae)
Source: Wellcome Open Res. 2026 Oct 23;11:908. [Version 1] doi: 10.12688/wellcomeopenres.28176.1 (PMC13728443; doi:10.12688/wellcomeopenres.28176.1)
Supplement: Peer review history

# Reviewer report: Reviewer response for version 1

**DOI:** 10.21956/wellcomeopenres.31001.r176206

**Published:** 2026-11-02

**Authors:** Joel F. Gibson

**Affiliations:** 1 Entomology, Royal BC Museum, Victoria, British Columbia, Canada

The present article is an excellent example of the use of modern genomic techniques to further the goals of Biodiversity science and Entomology in particular. The choice of a rare and likely endangered European species is the perfect use of this science. It is also valuable to increase the number of reference genomes for the Asilidae (and Diptera in general). The lab methods and bioinformatic techniques are up to date.

Are the datasets clearly presented in a usable and accessible format, and the assembly and annotation available in an appropriate subject-specific repository?

Yes

Are sufficient details of the sequencing and extraction, software used, and materials provided to allow replication by others?

Yes

Are the rationale for sequencing the genome and the species significance clearly described?

Yes

Are the protocols appropriate and is the work technically sound?

Yes

Reviewer Expertise:

Entomology, Diptera, Biodiversity, High Throughout Sequencing, bioinformatics

I confirm that I have read this submission and believe that I have an appropriate level of expertise to confirm that it is of an acceptable scientific standard.

# Reviewer report: Reviewer response for version 1

**Published:** 2026-10-23

**Authors:** Automated Benchmarking

**Affiliations:** 1 Wellcome Sanger Institute, Hinxton, UK

As this article is a Genome Note, Automated Benchmarking has been used to determine whether the reported assembly passes four defined checks established by the Earth BioGenome Project: the raw and assembled data are publicly available, the Earth BioGenome Project three-part metric is met, the k-mer completeness exceeds 90%, and the BUSCO score passes the stated threshold. For Cyrtopogon pulchripes (idCyrPulc1), all four checks passed. Data available: pass. ENA study PRJEB80967, raw-read accessions ERR13800504 and ERR13802646, and assembly accessions GCA_971800845.1 and GCA_971793335.1 are public. Earth BioGenome Project three-part metric: pass. Haplotype 1 is reported as 6.C.Q59, meeting the 6.C.Q40 reference standard. k-mer completeness: pass. The combined haplotype assemblies have 99.09% k-mer completeness, exceeding the Earth BioGenome Project target of 90%. BUSCO: pass. Haplotype 1, which is proposed as the reference assembly for this species, has 97.7% complete BUSCOs (95.8% single-copy and 1.9% duplicated). BUSCO software version: 6.0.0; lineage dataset: diptera_odb10. It meets the target of greater than 90% complete and single-copy BUSCOs. This decision uses values reported in the Genome Note and public accession records.

Reviewer Expertise:

NA

I confirm that I have read this submission and believe that I have an appropriate level of expertise to confirm that it is of an acceptable scientific standard.
